# A qualitative content analysis of "problem students": how can we identify and manage them?

**DOI:** 10.1186/s13104-020-05393-8

**Published:** 2020-12-17

**Authors:** Soleiman Ahmady, Nasrin Khajeali, Masomeh Kalantarion, Mitra Amini

**Affiliations:** 1 Department of Medical Education, Virtual School of Medical Education and Management, Shahid Beheshti University of Medical Sciences, Tehran, Iran; 2 Fasa University of Medical Sciences, Fasa, Iran; 3 Department of Medical Education, Students Research Committee, Virtual School of Medical Education and Management, Shahid Beheshti University of Medical Sciences, Tehran, Iran; 4 Clinical Education Research Center, Shiraz University of Medical Sciences, Shiraz, Iran

**Keywords:** Problem students, Medical student, Medical education

## Abstract

**Objectives:**

Problem students is one of the important issues in medical education. This study aimed to identify the problem students and the ways for managing these students from the educational experts view. Purposive sampling was used, and data collection continued until data saturation was achieved. Data analysis was performed by the content analysis method based on the Heidegger approach. We interviewed 12 educational experts who had a history of dealing with "problem students”.

**Results:**

After data analysis, five main themes and 28 categories, and 164 codes were extracted. The reasons for changing a student to a problem students was: student self-regulation skills, multilayer interactions, curriculum failure, identification policy and supportive solutions. The results indicated that despite revision in the curriculum, there were shortcomings in identification and management of problem students. According to participants, existence of a comprehensive system and a capable counseling center can identify the problem student sooner. On the other hand by improving self-regulation skills, active teaching methods and frequent formative evaluation and the use of supportive strategies, problem student can be encouraged to complete their education successfully. This study emphasized faculty development, reviewing the faculty member recruitment, strengthening counseling centers, improving the exams.

## Introduction

In some students, admission to the university causes cultural, social, and psychological deprivations and increases anxiety. In such cases, the student is unable to adapt efficiently and effectively and suffers from academic failure [1]. The academic decline is one of the problems of educational systems, including universities [2].

Vaughn et al. define a problem students as “a learner whose academic performance has declined significantly due to an emotional, cognitive, structural, or individual problem” [3]. It can ensue consequences such as addiction, apathy, anxiety, depression, and even suicide [2]. Problem students have various causes such as inappropriate teaching methods, improper use of teaching aids, the physical and social environment of the classroom, and the motivation of students and loneliness, family circumstances, and biological factors [4]. Also, students' academic failure is a major social problem, rather than a personal issue, which requires fundamental steps to solve [5, 6]. Meanwhile, academic decline is of particular importance in regard to medical students due to their job sensitivity and direct relationship with people’s health [6, 7].

Therefore, the problem students is of particular importance regarding medical students due to their job sensitivity and direct relationship with people's health [4, 7]. In these students, dropping out will further entail poor performance in hospitals and medical centers, sometimes leading to irreversible conditions. Due to the importance of identifying these students, and little evidence for identifying and providing support for them, we aimed to explore how to identify and manage problem students among the students of Shahid Beheshti University of Medical Sciences. To the best of our knowledge, no research has been conducted on this issue in Iran; therefore, this study aimed to identify and manage "problem students" in medical education.

The data collection was performed via a one-on-one semi-structured. Semi-structured interview allow participants to show their opinions in their own words freely. It can provide valid and reliable data.

## Main text

### Methods

This qualitative approach was designed and implemented by a content analysis method [8].

### Participants

The study population consisted of a director, experts in student support system, and the faculty members, who had a history of dealing with "problem students." Before starting the interview, the researcher built a good rapport with the participants and explained to the interviewees the purpose of the research. The study population consisted of director the Center for Education and Development of the Ministry of Health, the Deputy Minister of Education, the faculty members, a Faculty in Counseling Psychology, who had a history of dealing with “problem student”.

### Data gathering

A member of the research team (N Kh) who is also has a history of studying with problem students, interviewed. Researchers used purposive sampling to provide their expert opinion on problem identification and management. Sampling continued until data saturation, and 12 respondents entered the study. One participants dropped out of the study because of didn’t have time. Each interview conducted in a private room in the medical school or Counseling center. We gathered additional information regarding age and sex from participants. The mean age of those participants was 52 years (range 42–62 years) and 77% were male and 23% were female.

To begin exploring the expert’ experiences, pilot study was conducted. The interviews were recorded by a tape recorder to increase the accuracy of data collection. Interviews lasted between 30 and 60 min. Filed note didn’t used.

Semi-structured interviews were done based on general questions such as the following questions:

- Did you have any experience in dealing with problem students? How did you identify them? (May you define an objective example of coping with problem students?
- What are the barriers and problems for problem students? Can you talk more about this?
- In the current education system, what criteria (formal and informal) are problematic for comprehensive identification? By what criteria are they identified? What services do they receive?
- What happens to them in the end?
- What strategies are needed to be able to manage them?
- What strategies are required to be able to support them?

#### Data analysis

The data were analyzed by the inductive qualitative content method [10]. After each interview, the interviews were immediately transcribed. Then, the text of the meetings was reviewed to gain a general understanding. After that, each summarized unit was abstracted and named with a code by two researchers. The codes were categorized based on similarities and how to merge them. 12 interview texts were examined and confirmed by two researchers. We didn’t use software for analyzing.

#### Trustworthiness

Dependability, the codes were checked with participants, and the accuracy of the information was examined.

Guba and Lincoln's criteria were used to achieve credibility and dependability [4]. Credibility, the interpretation and report (or a portion of it) were given to the members of the sample (informants) to check the authenticity of the work.

Furthermore, the findings were verified by external auditors familiar with qualitative research. It means that parts of the interview text, along with the relevant codes and classes produced by the two observer’s casual with qualitative research, were examined and confirmed. To make the findings transferable, we tried to transcribe the participants' sentences verbatim.

#### Ethical considerations

Participant's information was kept confidential to the researcher, and individuals had the option to withdraw from the study at any stage of the research.

### Results

Five main themes emerged in this study: self-regulation skills, multilayer interactions, curriculum failure, identification policy, and supportive solutions.

#### Self-regulation skills

This theme containing three more related categories including: self-awareness, low goal setting, and inability to describe oneself without judgment.

One participant ascertained: I think one of the significant challenges is that they don't have self-awareness, and that's why they're justifying it instead of rooting the problem out.

#### Multilayer interactions

This theme refers to the four categories: family role, peer role, hidden curriculum, and teacher-student relationships.

Family members both positively and negatively contribute to the prevention or creation of problematic students.

Participants said in this regard: "We have another problem. There are parents, called helicopter parents, according to our field. Helicopter parents are those who raise their children under the age of 18 in the lap of luxury. Then, they immediately bring their children to university and throw them down. They're spinning up. They're neither going to take our hand nor helping us to see what we should do. The story of our intervention is that the family has to intervene, but they are inefficient".

Participants in the study reported that the hidden curriculum and teacher-student relationships also played an essential role in creating or preventing problem learners.

#### Curriculum failure

The most common codes are presented in this theme. This could be described by three categories: inappropriate curriculum, ineffective teaching, and evaluation are the causes of curriculum failure.

One of the respondents said: teachers' inability to effectively communicate when teaching led to problem students.

A participant commented about the inappropriate curriculum: "For example, our problem is about the expected curriculum. Some students believe that the content of the medical curriculum is irrelevant to their future job needs. Hence, differences between the expected and experienced curricula sometimes make a huge discrepancy that can lead to disappointed students.

#### Identification policy

In this study, it has been stated that the identification system isn't systematic.

Some participants reported: some institutions identified a problem student based on formal criteria, while others identified informal group meetings, so there was no comprehensive identification policy.

#### Supportive strategies

In this category, supportive strategies have been proposed based on the role of family, peers, educational system, and teachers.

One participant said:"

In the educational system, in addition to assessing knowledge, we measure items such as study skills, learning style, etc., it can help us not have problem students.

Another participant reported:

The failure is desirable in some cases when the individuals have no talent in a particular field. So, these failures warn us to encourage the student to choose another field.

Some related themes and quotes are listed in the Table 1.

**Table 1:** Some related themes and quotes

Theme	Quotations
Self-regulation skills	Nothing terrible happens to students because they don't realize at all. One skill is self-awareness—the ability to look at oneself
Multilayer interactions	Our teachers are generation x, students of generation y and z, and this difference between the generations causes them not to read each other's words
Curriculum failure	Evaluation should be tailored to the purposes of teaching. If our test isn’t in line with the objectives of the lesson, it causes confused student and reduce her interest
Identification policy	Usually, identifying a problem student in the educational system is through criteria, but it still does not do well, that is, the same things that are mentioned in the regulations are not implemented
Supportive strategies	Now one of the arguments is to reduce the number of summative assessments, increase the number of formative assessments

### Discussion

This study aimed to explain the understanding of experts regarding the identification and management of problem medical students.

Self-regulation skill is the first theme of this study. Participants reported this skill is one of necessary skills for medical students. Guntern stated a significant relationship between self-regulatory skill and academic achievement [9].

Sobral [10] also showed that the more learning leads to reflective learning, the better one's academic achievement and preventing problem students. Reflective thinking, which is one of the sub-categories of self-regulatory skills [10].

Multilayer interactions was another theme in our study. Steinert points to the critical role of classmates and teachers in identifying and helping problem students [2]. If the teacher-student relationship is well established, educational goals will be achieved with more quality and ease. That was similar to our study.

In a case report of a dismissed medical student with a higher educational background, Aghaei Afshar emphasizes the role of the family in paying attention to their children's abilities and interests in identifying a problem student. On the other hand, the university counseling system should identify the endangered students especially at the beginning of the study and take care of them with maintain contact with the family [11].

Another important finding of the current study was the theme of curriculum failure in higher education. Participants cited the inappropriate curriculum, ineffective assessments, and lack of effective teaching methods as factors of curriculum failure. These results are consistent with Roos's study [12]. Therefore, curriculum should be tailored to the needs of the community, so students can provide valuable insights for the curriculum and this has impact on the learning process, which is essential for educational centers.

In the study of the graduates, one of the graduates was asked about the extent of the curriculum presented, only 4% agreed with the appropriateness of the curriculum to empower the physicians [13].

Regarding the theme of identification policy, the participants pointed out the lack of a comprehensive system of identification and ineffective intervention, consistent with the results of Shams [14]. Therefore, a global identification system and effective response are required to support problem students.

The last theme was about supportive strategies. In this regard, Students can be supported through family, peers, educational systems, and teachers. These strategies have been reported in Steiner's study [2].

Aghaei Afshar is highlighted in family’s role in identifying problem student in case of dismissed medical student [11].

The result of our study was in line with the results of other studies and had a more comprehensive view of problem student identification in comparison to previous studies because it examined various aspects of medical education such as curriculum planning, teaching methods, evaluation, support strategies and educational policy changes.

The present study emphasized that we could identify and manage the problem students with the best approach by faculty development, reviewing the faculty member recruitment, strengthening counseling centers, improving the exams, and screening the students on arrival.

Future research could focus on recognize the demographic psychological status of problem learner for identifying and increase their coping rate with effective interventions. We designed a qualitative study, so did not investigate the files of the non-problem students in the cohorts. So a full case–control study would be required.

### Limitations

A limitation of the present study was that some participants did not allow to record conversations; hence, the researcher had to write down everything she heard. Another limitation was that the researcher made several appointments to interview the experts, but the interviews were postponed due to the professors' busy schedule.

## Data Availability

The datasets used and analyzed during the current study are available from the corresponding author on reasonable request.
